# Identification of 6 cuproptosis-related genes for active ulcerative colitis with both diagnostic and therapeutic values

**DOI:** 10.1097/MD.0000000000035503

**Published:** 2023-10-27

**Authors:** Menglong Zou, Wei Zhang, Ying Zhu, Yin Xu

**Affiliations:** a The First Hospital of Hunan University of Chinese Medicine, Changsha, Hunan, China; b College of Chinese Medicine, Hunan University of Chinese Medicine, Changsha, Hunan, China.

**Keywords:** biomarkers, cuproptosis, machine learning, pharmacology, ulcerative colitis

## Abstract

Cuproptosis has been reported to affect a variety of diseases. Therefore, we aimed to examine the role of cuproptosis-related genes in active ulcerative colitis (UC). We acquired 2 datasets of active UC from the Gene Expression Omnibus database and created immune cell infiltrations to research immune cell dysregulation. Based on the cuproptosis gene set and differentially expressed genes (DEGs), we identified the differentially expressed genes of cuproptosis (CuDEGs). We then used 2 machine learning methods to screen hub CuDEGs. Subsequently, we performed validation on additional datasets and investigated the relationship between hub CuDEGs and drug treatments. Thirty-five controls with inactive UC and 90 patients with active UC were obtained from the training sets. A total of 9157 DEGs and 27 CuDEGs were identified, respectively. Immune cell infiltration analysis revealed that patients with active UC exhibited higher levels of activated dendritic cells and neutrophils as well as lower levels of CD8+ T cells, regulatory T cells (Tregs), and macrophage M2. A six-gene cuproptosis signature was identified using machine learning algorithms. We further validated that the 6 hub CuDEGs showed a strong correlation with active UC and acted as cuproptosis-related biomarkers of active UC. Moreover, the expression of ATOX1 was downregulated, and SUMF1, MT1G, ATP7B, FDX1, and LIAS expression was upregulated in the colonic mucosa of active UC patients who responded to golimumab or vedolizumab therapy. With the exception of ATP7B, the expression patterns of hub CuDEGs before and after infliximab treatment of patients with active UC were similar to those of golimumab and vedolizumab. Cuproptosis and active UC have a complex relationship, as illustrated in our study. ATOX1, SUMF1, MT1G, ATP7B, FDX1, and LIAS are cuproptosis-related hub genes of active UC. Our study opens new avenues for investigating UC progression and developing novel therapeutic potential targets for the disease.

## 1. Introduction

Ulcerative colitis (UC) is a chronic, recurrent, immune-mediated inflammatory disease that affects the mucosa of the rectum and colon.^[1]^ A number of factors contribute to the occurrence and progression of UC, including immune disorders, destruction of barrier function, abnormal cytokine production, and intestinal microbiota.^[2,3]^ The UC classification is critical for clinical management because of the heterogeneity of the patient population.^[4,5]^ Gene expression profiling using high-throughput microarrays has enabled the identification of genes associated with the clinical phenotypes.^[6,7]^ Several genes associated with UC phenotypes have recently been identified as biomarkers, but the evidence may not be convincing due to the limited sample size or single dataset.^[8,9]^ Hence, accurate identification of UC-related subtypes at the molecular level is crucial for disease treatment and management.

Copper ions are enzyme cofactors whose homeostasis is controlled primarily by the mitochondria.^[10]^ Many biological processes, including cell redox homeostasis, rely on copper, a component of superoxide dismutase and cytochrome C oxidase.^[11,12]^ It has been shown that copper homeostasis disorders are closely linked with UC.^[13]^ Notably, the presence of elevated copper levels within cells also promotes the development of colitis in colon cancer.^[14]^ Therefore, it is rational to assume that intracellular copper contributes to the development of UC. Cuproptosis is a novel mechanism of cell death mediated by copper toxicity compared with other forms of regulated cell death.^[15]^ Copper serves both as a necessary component and has potential toxicity in cells.^[16]^ Living organisms require copper as an essential cofactor to function properly, but high levels of copper accumulation can cause cell death.^[17,18]^ Cuproptosis, characterized by the excessive accumulation of lipidated mitochondrial enzymes, is closely linked to mitochondrial stress.^[19]^ Numerous studies have revealed that mitochondrial dysfunction plays an important role in UC.^[20–23]^ Consequently, we can further infer that cuproptosis is strongly linked to UC progression. However, there is a lack of knowledge regarding the mechanisms underlying cuproptosis in the progression of UC.

A systematic comparison of cuproptosis-related genes (CRGs) between patients with active and inactive UC was conducted for the first time in the current report. Subsequently, learning algorithms were employed to screen the hub CRGs. We examined the relationship between hub CRGs and the treatment effects of drugs currently commonly used in active UC, such as golimumab (GLM), infliximab (IFX), and vedolizumab (VDZ). This will contribute to the elucidation of the pharmacological mechanisms and optimization of individualized treatment of active UC.

## 2. Materials and Methods

### 2.1. Datasets and preprocessing

Data sets for active UC were obtained from the Gene Expression Omnibus database. Table 1 provides a description of the information for all datasets in our study. MINiML files were used for raw data download. Data from MINiML files, including platform, sample, and GSE records, were extracted, and the resulting data were log2 transformed for standardization. A batch effect between different data sets was removed using the “limma” package (version 3.52.4) in the R software (version 4.2.0). The batch correction results were verified using principal component analysis.

**Table 1:** The platform information corresponding to all data sets in the present study.

Database	Platform	Title
GSE53306	GPL14951	Illumina HumanHT-12 WG-DASL V4.0 R2 expression beadchip
GSE75214	GPL6244	[HuGene-1_0-st] Affymetrix Human Gene 1.0 ST Array [transcript (gene) version]
GSE59071	GPL6244	[HuGene-1_0-st] Affymetrix Human Gene 1.0 ST Array [transcript (gene) version]
GSE107499	GPL15207	[PrimeView] Affymetrix Human Gene Expression Array
GSE92415	GPL13158	[HT_HG-U133_Plus_PM] Affymetrix HT HG-U133 + PM Array Plate
GSE73661	GPL6244	[HuGene-1_0-st] Affymetrix Human Gene 1.0 ST Array

CRGs were obtained from the MsigDB database and from previous studies.^[15,19,24,25]^ After removing duplicates, 57 genes associated with cuproptosis were identified. Ethical approval was not required for this study because it was a bioinformatics analysis.

### 2.2. Analysis of immune cell infiltration

Based on gene expression data obtained from each sample, the relative abundances of 22 immune cells in colon tissue were inferred using the CIBERSORT algorithm (http://cibersortx.stanford.edu). For each sample, CIBERSORT obtains a *P*-value based on the Monte Carlo sampling. To accurately determine the immune cell fraction, samples with *P* values <.05 were considered. In each sample, 22 immune cell proportions totaled one.

### 2.3. Differential expression analysis

We set *P* < .05 as the threshold to screen differentially expressed genes (DEGs) between active UC and inactive UC samples.

### 2.4. Enrichment analysis

To better elucidate the potential pathological mechanisms of active UC, R package “clusterProfiler” (version 4.4.4) was used to perform Gene Ontology (GO) and Kyoto Encyclopedia of Genes and Genomes analyses based on DEGs.

### 2.5. Machine learning

Overlapping genes were defined as differentially expressed genes of cuproptosis (CuDEGs) by intersecting cuproptosis-related genes and DEGs. Two machine-learning algorithms were used to further screen the diagnostic genes for active UC. The least absolute shrinkage and selection operator (LASSO) algorithm is a regression model that allows for both feature selection and regularization. A support vector machine (SVM) is a powerful approach with the goal of creating a threshold between 2 categories, which allows model prediction based on either single or multiple feature vectors. We used the “glmnet” package (version 4.1.6) and the “kernlab” package (version 0.9.31) to conduct the LASSO regression and SVM analysis, respectively. The overlapping genes of the 2 machine learning algorithms were considered hub CuDEGs for active UC diagnosis. ROC analysis was conducted to further assess the diagnostic value of active UC using the “pROC” package (version 1.18.0).

### 2.6. Statistical analysis

All data are presented as mean ± SE. Unpaired Student *t* test was used to compare 2 groups, and one-way ANOVA was used for comparisons between 3 or more groups. *P* < .05 was considered statistically significant.

## 3. Results

### 3.1. Identification of DEGs

**Figure 1.**
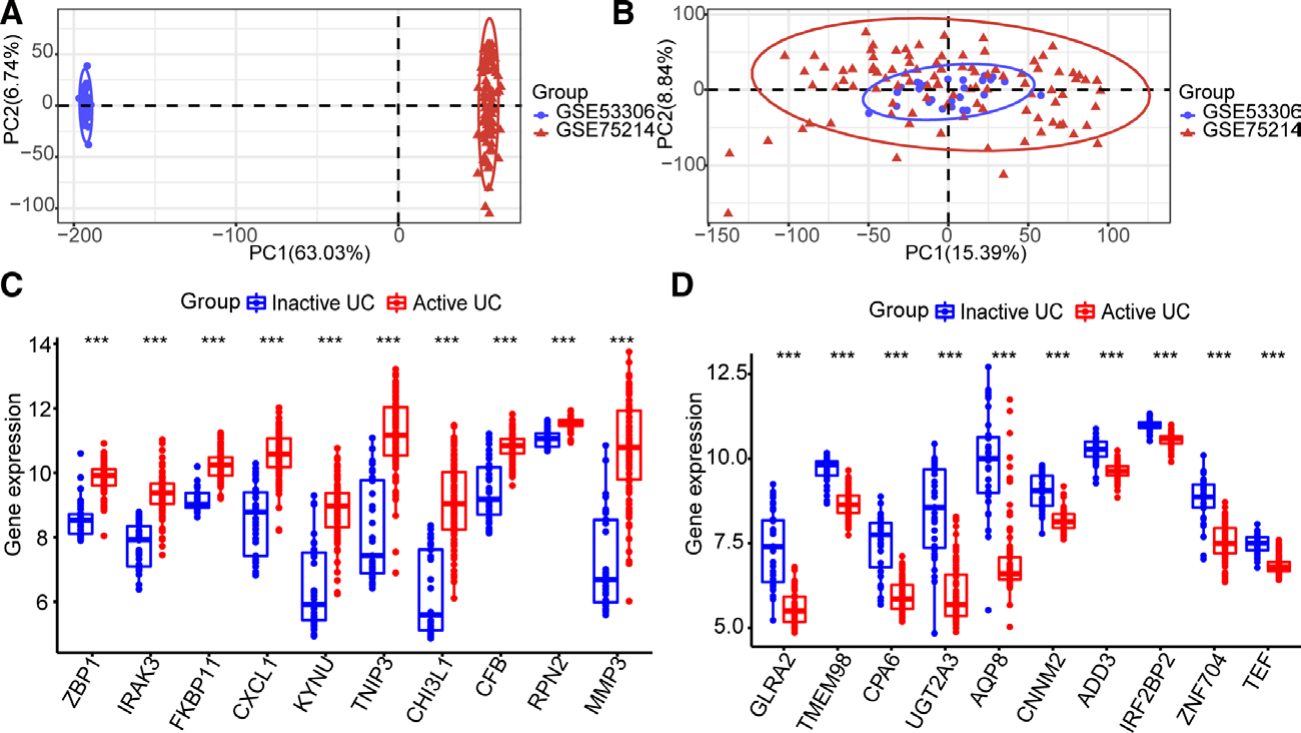
Identification of differentially expressed genes between active UC and inactive UC. PCA before (A) and after (B) batch correction for all samples. (C) Top 10 up-regulated genes. (D) Top 10 down-regulated genes. PCA = principal component analysis, UC= ulcerative colitis.

Based on the principal component analysis results, it was determined that the batch effect between the 2 datasets was eliminated (Fig. 1A and B). Nine thousand one hundred fifty-seven DEGs were identified, and the top 10 significantly differentially expressed genes are shown in Figure 1C and D.

### 3.2. Enrichment analysis of DEGs

**Figure 2.**
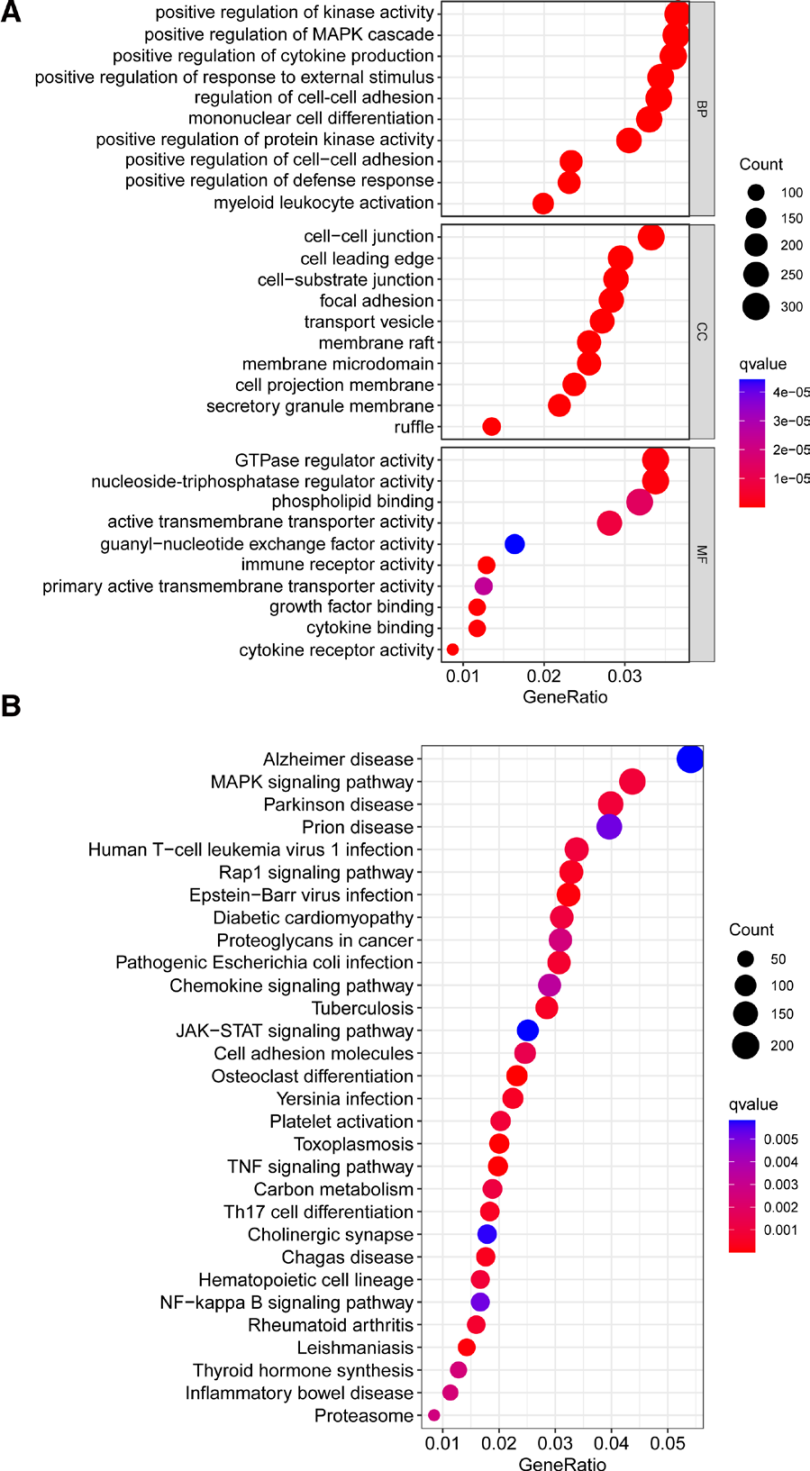
Enriched items in GO and KEGG analyses using DEGs. (A) Enriched items in GO analysis. (B) Enriched items in KEGG pathway analysis. DEGs= differentially expressed genes, GO= Gene Ontology, KEGG = Kyoto Encyclopedia of Genes and Genomes.

We used the R software to conduct enrichment analysis to explore the possible roles of these DEGs. As illustrated in Figure 2A, GO enrichment analysis showed that positive regulation of kinase activity, positive regulation of the MAPK cascade, positive regulation of cytokine production, cell–cell junction, cell leading edge, cell–substrate junction, GTPase regulator activity, nucleoside-triphosphatase regulator activity, and phospholipid binding were significantly enriched. Kyoto Encyclopedia of Genes and Genomes enrichment analysis revealed that MAPK, Rap1, JAK-STAT, and TNF are important pathways during UC development (Fig. 2B).

### 3.3. Evaluation of immune cell infiltration

**Figure 3.**
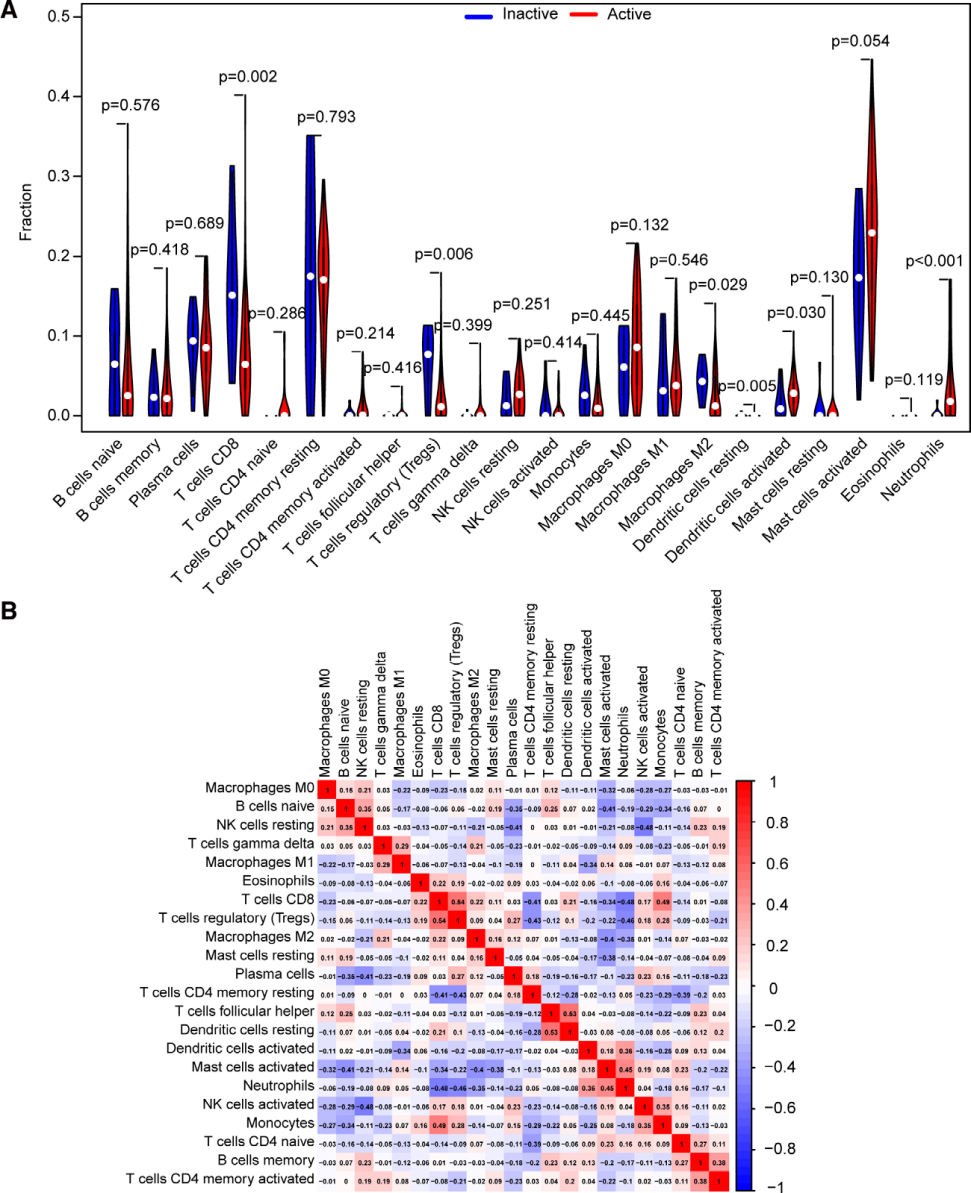
Immune cell infiltration in patients with active UC. (A) Violin plot of the differences in immune infiltration based on the inference of CIBERSORT algorithm between inactive UC and active UC. (B) Comparison of 22 immune cell subtypes between patients in inactive UC and active UC. UC= ulcerative colitis.

UC is characterized by a chronic inflammatory intestinal barrier, and dysregulation of mucosal immunity may be involved in the progression of UC. Hence, we investigated the differences in 22 immune cell infiltrations using the CIBERSORT algorithm (Fig. 3A and B). The boxplot shows that patients with active UC exhibited higher levels of activated dendritic cells (*P* = .030) and neutrophils (*P* < .001), as well as lower levels of CD8+ T cells (*P* = .002), regulatory T cells (Tregs) (*P* = .006), and macrophage M2 (*P* = .029). In addition, there was a weak association between infiltrating immune cells (Fig. 3B). CD8+ T cells were slightly more closely related to other immune cells. It had a weak negative correlation with neutrophils (r = −0.48) and CD4 memory resting T cells (r = −0.41) and a weak positive correlation with T cells regulatory (Tregs) (*R* = 0.54) and monocytes (*R* = 0.49).

### 3.4. Identification of hub CuDEGs

**Figure 4.**
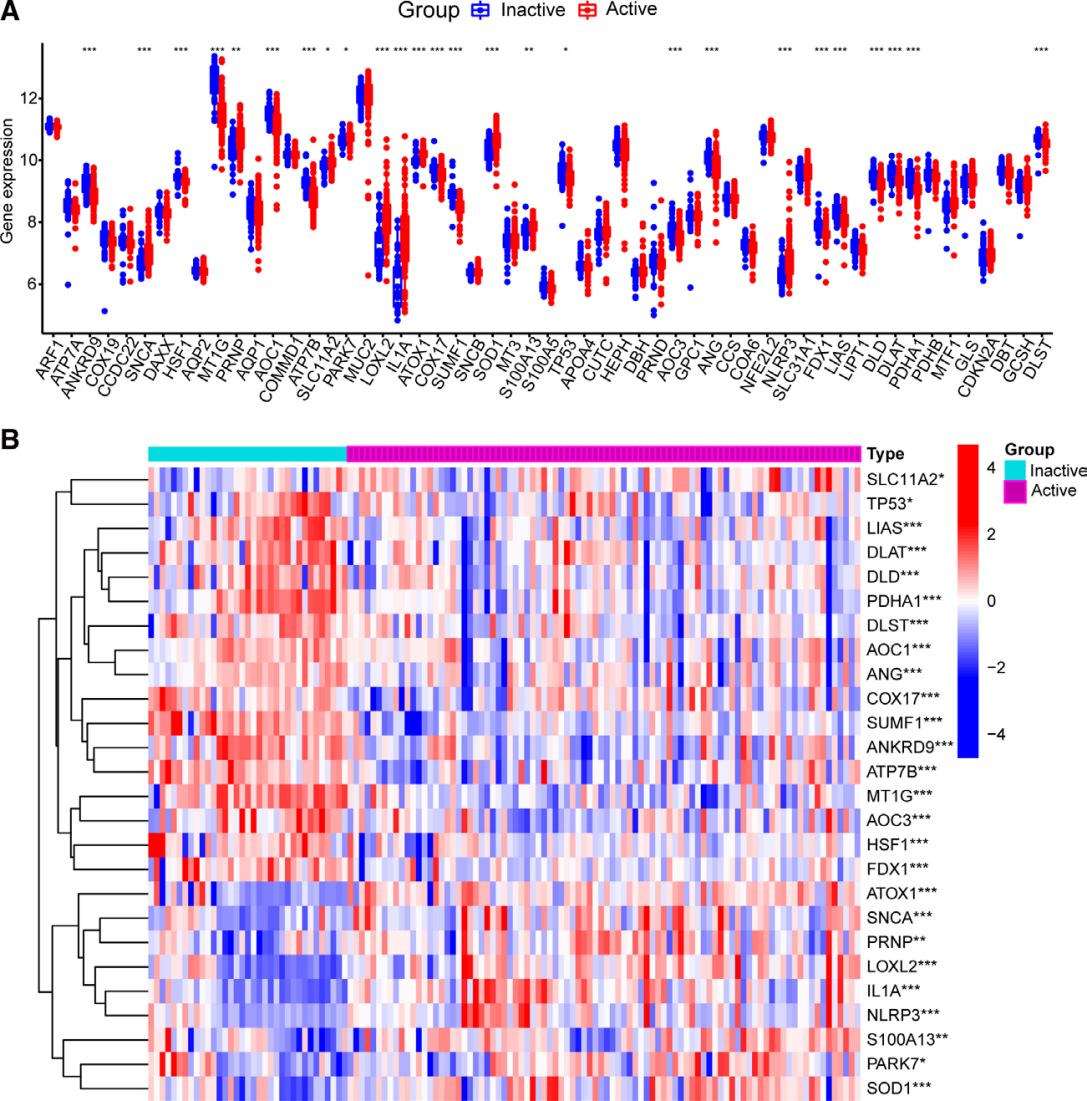
Screening of the CuDEGs in CD. (A) Boxplots of CuDEGs expression levels between inactive UC and active UC. (B) Heatmap of CuDEGs expression levels between inactive UC and active UC. *P < .05, **P < .01, ***P < .001. CuDEGs= differentially expressed genes of cuproptosis, UC= ulcerative colitis.

**Figure 5.**
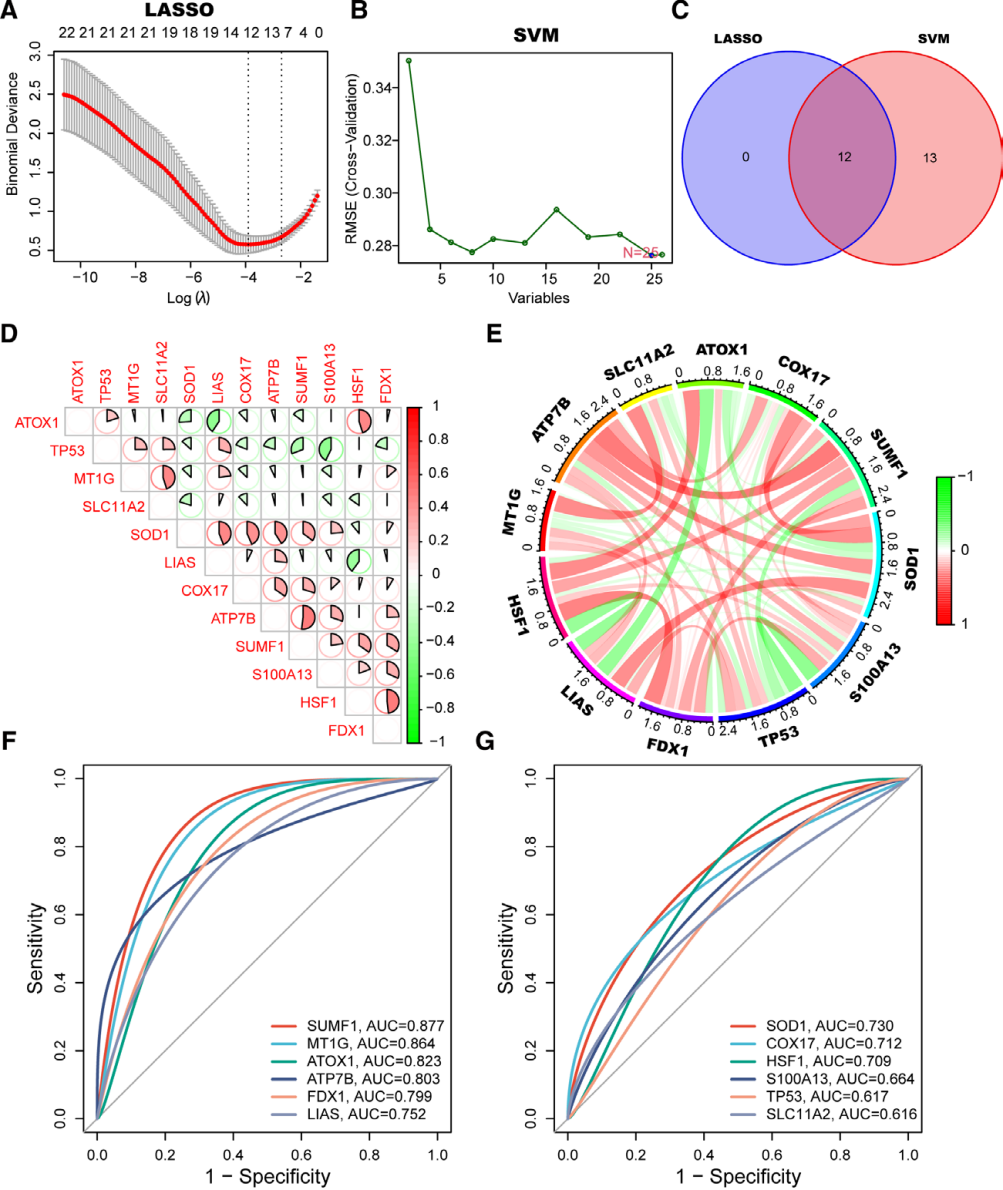
Machine learning in the identification of cuproptosis-signature. (A and B) Construction of cuproptosis-signatures using LASSO regression and SVM algorithm. (C) The Venn diagram shows the overlap of candidate genes between the above 2 algorithms. (D) Correlation analysis of the overlapping cuproptosis genes. (E) Circos plot of shows the relationship between the overlapping cuproptosis genes. (F and G) ROC curve of cuproptosis-signatures in active UC diagnosis. LASSO= least absolute shrinkage and selection operator, SVM= support vector machine, UC= ulcerative colitis.

To analyze the role of cuproptosis in UC progression, we initially examined the differential expression of 57 CRGs in active and inactive UC. The total number of CuDEGs identified was 26. The expression levels of ATOX1, SNCA, PRNP, LOXL2, IL1A, NLRP3, S100A13, PARK7, and SOD1 were higher, whereas those of SLC11A2, TP53, LIAS, DLAT, DLD, PDHA1, DLST, AOC1, ANG, COX17, SUMF1, ANKRD9, ATP7B, MT1G, AOC3, HSF1, and FDX1 were lower in active UC samples than in inactive UC samples (Fig. 4A and B). After obtaining the CuDEGs, the SVM and LASSO algorithms were used to filter hub genes for the construction of the cuproptosis-signature (Fig. 5A and B). Ultimately, 12 CRGs were obtained (Fig. 5C). To investigate the role of cuproptosis regulators in UC progression, we conducted correlation analysis between these 12 genes. Surprisingly, some cuproptosis-signature genes such as ATP7B and SUMF1 exhibited a significant synergistic effect. In parallel, ATOX1 and LIAS appeared to be antagonistic (Fig. 5D). Further evidence for the close relationship between cuproptosis-signature genes was observed in the gene relationship network diagram (Fig. 5E). To assess the diagnostic value, ROC curves of the 12 gene signatures were analyzed (Fig. 5F and G). To achieve more reliable diagnostic values, we screened 6 hub genes with AUC values >0.75 for further analysis (Fig. 5F). The AUC for SUMF1, MT1G, ATOX1, ATP7B, FDX1, and LIAS were 0.877, 0.864, 0.823, 0.803, 0.799, and 0.752, respectively.

### 3.5. The relationship of 6 hub CuDEGs with immune cells

**Figure 6.**
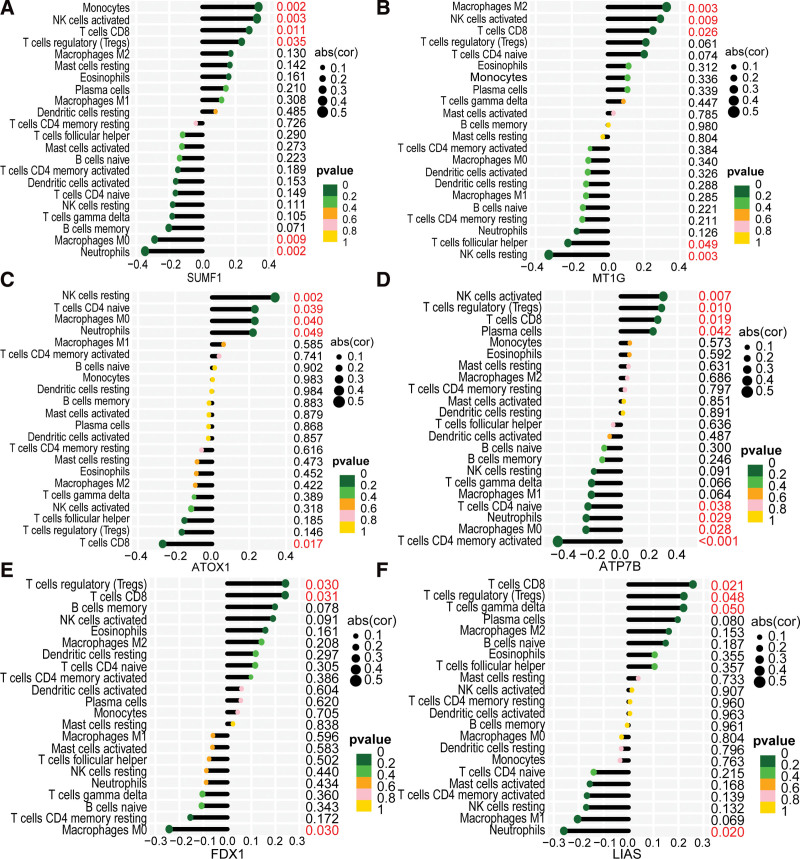
Correlation between immune infiltrating cells with SUMF1, MT1G, ATOX1, ATP7B, FDX1, and LIAS (A–F).

We conducted a correlation analysis to determine whether the 6 diagnostic genes were associated with infiltrating immune cells in active UC (Fig. 6A–F). Interestingly, 5 diagnostic genes, including SUMF1, MT1G, ATP7B, FDX1, and LIAS, exhibited a significantly positive relationship with CD8+ T cell infiltration, and one diagnostic gene, ATOX1, had a significantly negative relationship with CD8+ T cell infiltration.

### 3.6. Verification of hub CuDEGs

**Figure 7.**
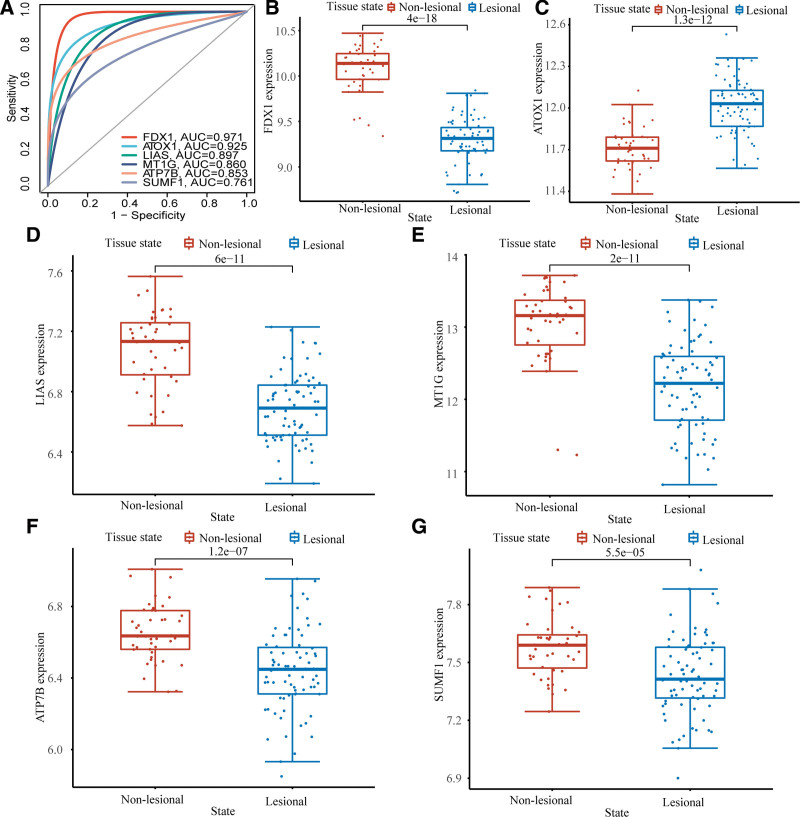
Verification of cuproptosis-related hub genes in GSE107499. (A) ROC curve of cuproptosis-related hub genes in active UC diagnosis. The expression of FDX1 (B), ATOX1 (C), LIAS (D), MT1G (E), ATP7B (F), and SUMF1 (G) in the lesional and non-lesional intestinal mucosa of active UC patients. UC= ulcerative colitis.

**Figure 8.**
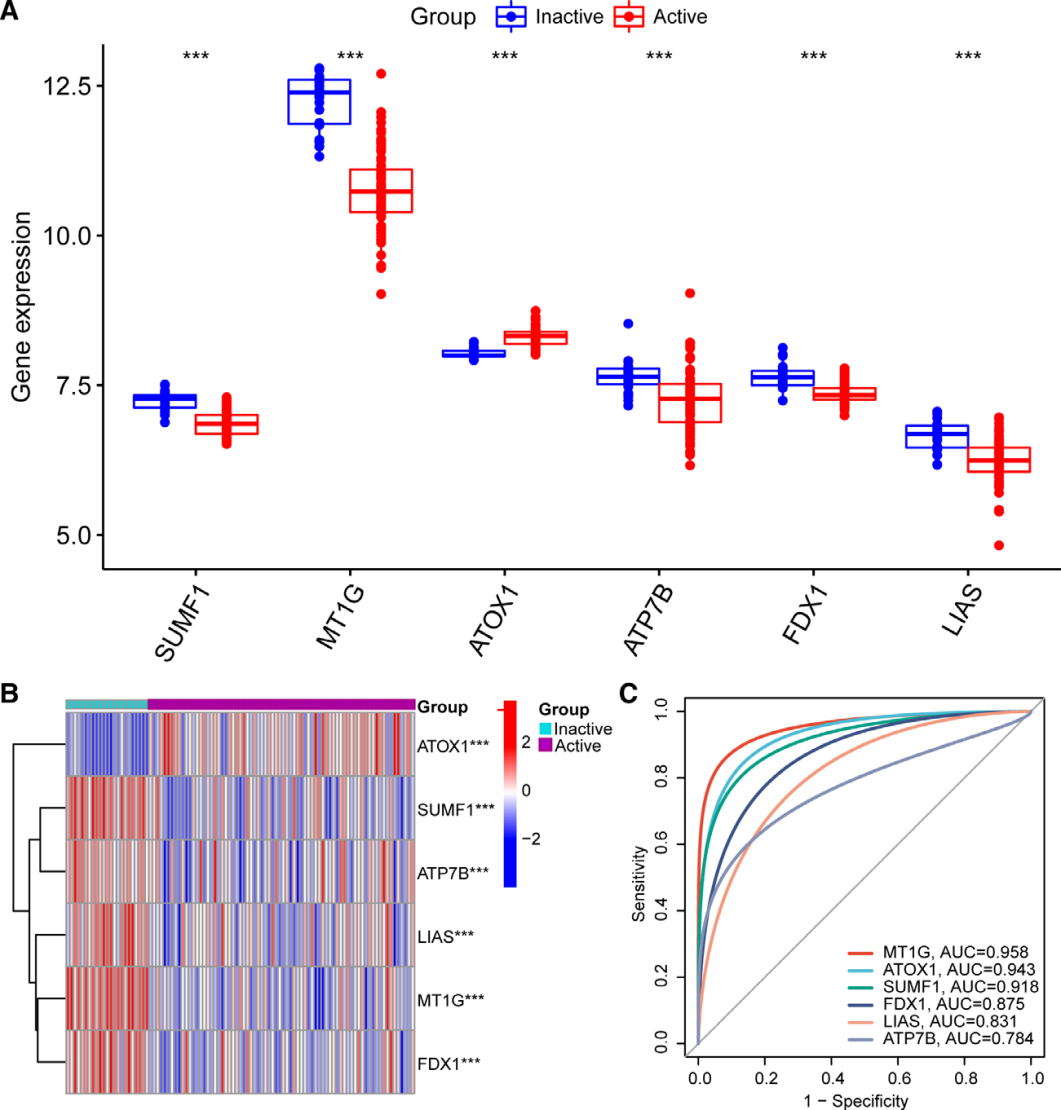
Verification of cuproptosis-related hub genes in GSE59071. (A) Boxplots of expression levels of cuproptosis-related hub genes between inactive UC and active UC. ***P < .001. (B) Heatmap of expression levels of cuproptosis-related hub genes between inactive UC and active UC. ***P < .001. (C) ROC curve of cuproptosis-related hub genes in active UC diagnosis. UC= ulcerative colitis.

We then verified the expression of the 6 hub CuDEGs using 2 additional datasets (GSE107499 and GSE59071). GSE107499 includes gene expression data from the lesional and non-lesional colon tissues of patients with active UC. The AUC values for FDX1, ATOX1, LIAS, MT1G, ATP7B, and SUMF1 were 0.971, 0.925, 0.897, 0.860, 0.853, and 0.761, respectively, indicating that the 6 hub CuDEGs also had excellent diagnostic values in GSE107499 (Fig. 7A). Figure 7B–G revealed that FDX1 (*P* = 4e−18), LIAS (*P* = 6e−11), MT1G (*P* = 2e−11), ATP7B (*P* = 1.2e−07), and SUMF1 (*P* = 5.5e−05) were downregulated and ATOX1(*P* = 1.3e−12) was upregulated in the lesion tissue in GSE107499. Furthermore, we analyzed the expression levels of these 6 hub CuDEGs in patients with active UC and those with inactive UC in GSE59071 and found a similar result (Fig. 8A and B). It is worth noting that the AUC values for all 6 hub CuDEGs were >0.75 (Fig. 8C).

### 3.7. Drugs improve colonic mucosal injury in patients with active UC by regulating CuDEGs

**Figure 9.**
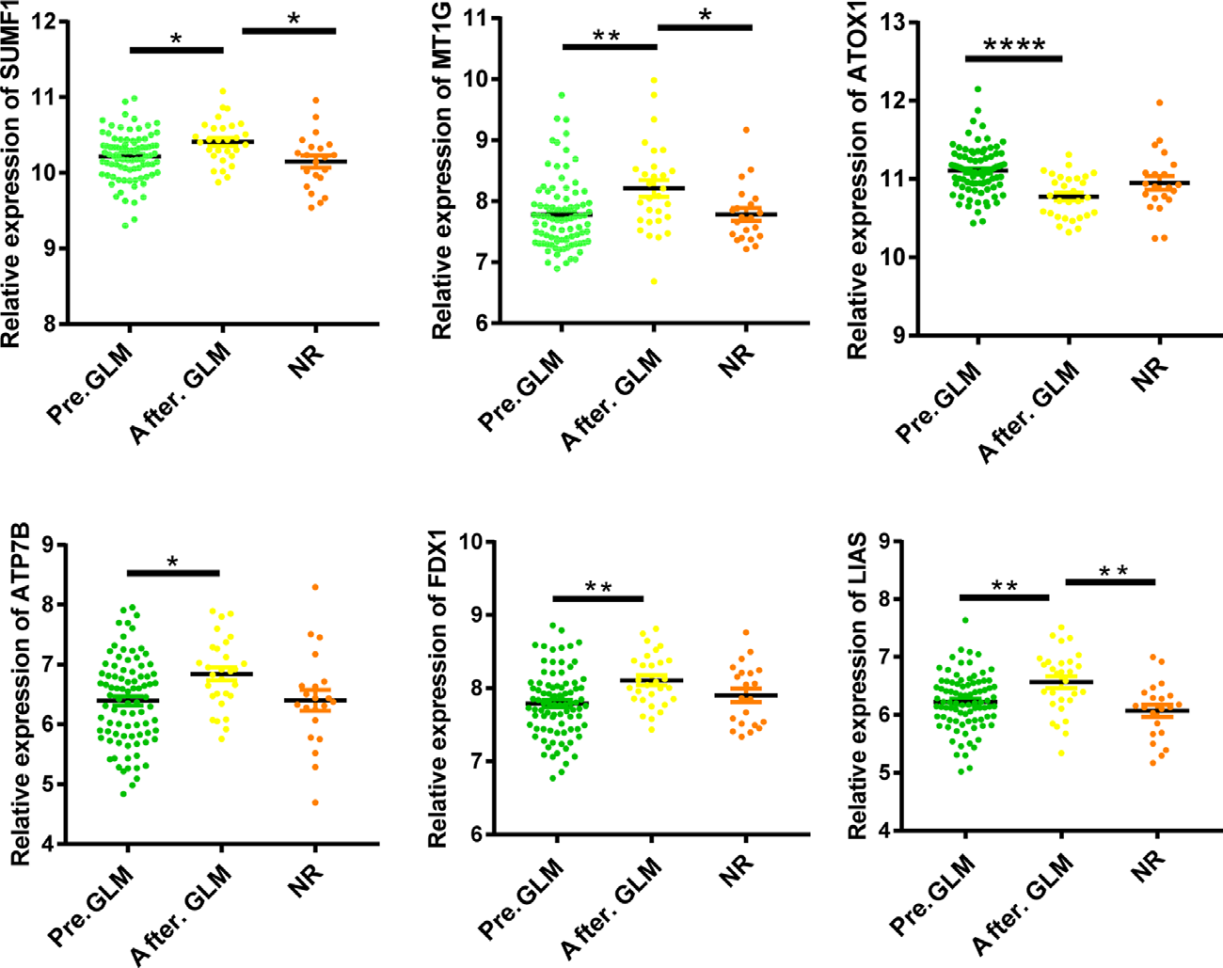
Golimumab responders improve impaired cuproptosis of intestinal mucosal in patients with active UC by regulating hub CuDEGs. *P < .05, **P < .01, ***P < .001. CuDEGs= differentially expressed genes of cuproptosis, UC= ulcerative colitis.

**Figure 10.**
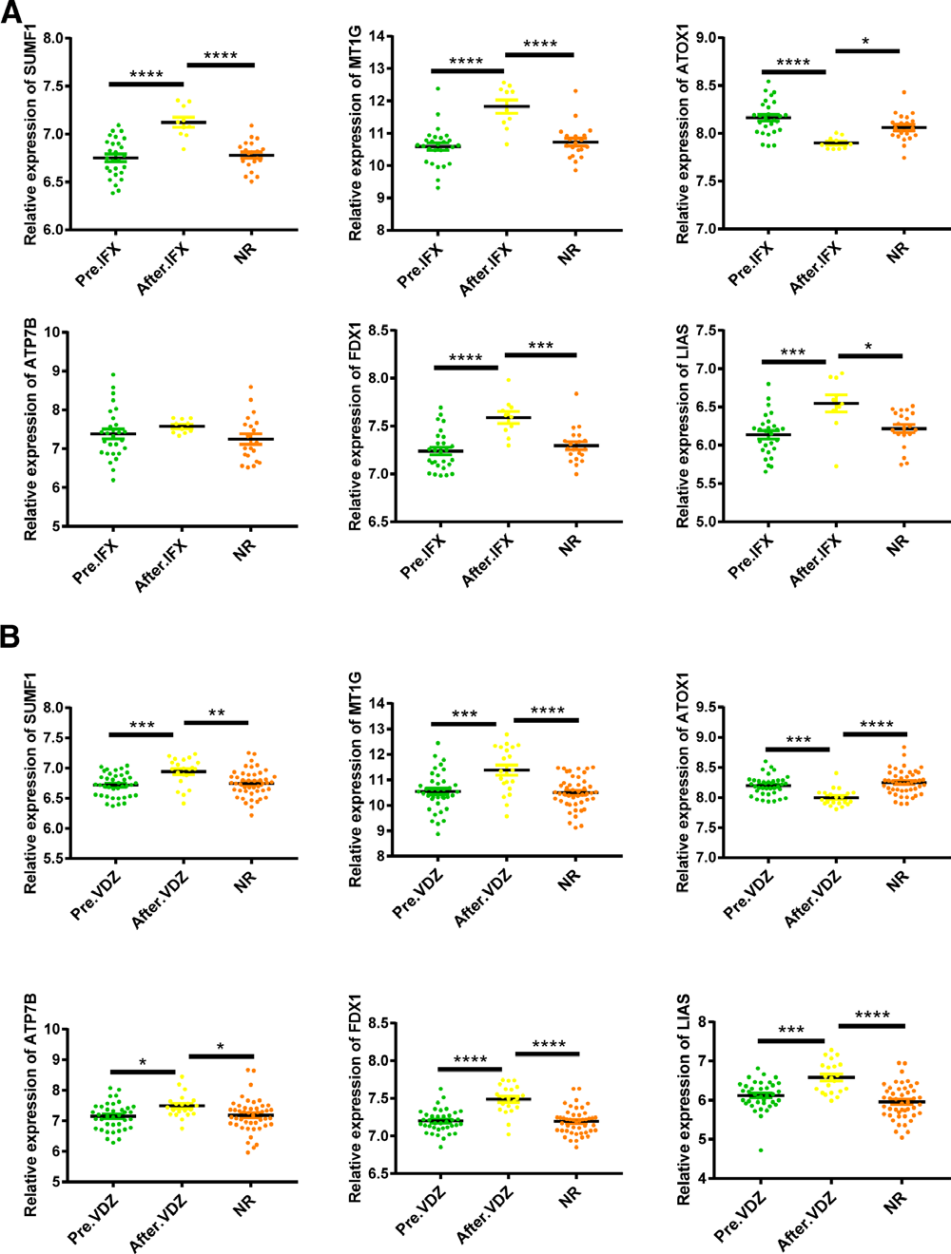
IFX and VDZ responders improve impaired cuproptosis of intestinal mucosal in patients with active UC by regulating hub CuDEGs. (A) The relative expression levels of SUMF1, MT1G, ATOX1, ATP7B, FDX1, and LIAS in the colonic mucosa of Pre.IFX (UC patients before IFX therapy), After.IFX (UC patients in remission after IFX therapy), and NR (UC patients not responding to IFX therapy). *P < .05, **P < .01, ***P < .001. (B) The relative expression levels of SUMF1, MT1G, ATOX1, ATP7B, FDX1, and LIAS in the colonic mucosa of Pre.VDZ (UC patients before VDZ therapy), After.VDZ (UC patients in remission after VDZ therapy), and NR (UC patients not responding to VDZ therapy). *P < .05, **P < .01, ***P < .001. CuDEGs= differentially expressed genes of cuproptosis, IFX= infliximab, VDZ = vedolizumab.

GLM, an anti-TNF-α monoclonal antibody, was approved by the Food and Drug Administration (FDA) for clinical use in UC in 2013.^[26]^ Subcutaneous injections of GLM promote colonic mucosal repair in patients with active UC. Our results revealed that the expression of all hub CuDEGs in patients with active UC was significantly changed after GLM treatment in GSE92415 (Fig. 9). IFX, which targets TNF-α, and VDZ, which targets α4β7, are recommended as first-line therapeutic agents for patients with moderate to severe UC. Our study revealed that the expression of ATOX1 was downregulated, and that of SUMF1, MT1G, FDX1, and LIAS was upregulated in the colonic mucosa of patients with active UC after IFX treatment in GSE73661 (Fig. 10A). The expression patterns of hub CuDEGs after VDZ treatment were similar to those of the GLM in GSE73661 (Fig. 10B).

## 4. Discussion

There have been significant advances in immunotherapy for UC over the past few decades, although the causes of the absence of immune responses in some patients are not known.^[27,28]^ Recently, copper-dependent cell death, also known as cuproptosis, has been linked to disease progression.^[29–31]^ However, there have been few studies on the specific mechanisms of cuproptosis in relation to active UC and its regulatory role has not yet been clarified. Therefore, we systematically analyzed the role of CRGs in active UC and the immunological microenvironment to elucidate the reasons for the lack of an immune response.

This study is the first to investigate the expression of CRGs in patients with active UC. We identified 26 dysregulated CRGs in active UC, suggesting that CRGs are critical for its progression. Next, 2 machine learning classifiers were used to select 12 hub genes. To further elucidate the connection between cuproptosis regulators and active UC, we determined the correlation between the 12 hub genes. The interaction of CRGs in patients with active UC confirmed that some CRGs have antagonistic or synergistic effects. We screened 6 hub CuDEGs (SUMF1, MT1G, ATOX1, ATP7B, FDX1, and LIAS) based on their AUC values. 80–90% of patients with UC alternate repeatedly between the active and inactive phases.^[32]^ The significant difference between the active and inactive phases of the hub CuDEGs demonstrated that upregulation or downregulation of hub CuDEG expression in patients leads to cuproptosis-induced intestinal mucosal damage and elevated pro-inflammatory factors in intestinal epithelial cells, resulting in disease recurrence. Therefore, focusing on the development of cuproptosis may be a novel approach to avoid the recurrence of active UC and an important future therapeutic direction. SUMF1 is responsible for activating all posttranslational modifications of sulfate esterases, whose deficiency leads to the accumulation of sulfated sugars, steroids, and lipids.^[33]^ Upregulation of MT1M has been reported to inhibit RGC cell inflammation and apoptosis.^[34]^ ATOX1 captures cytosolic copper and subsequently transfers it to copper pumps in the trans-Golgi network, thereby facilitating copper availability to various copper-dependent oxidoreductases.^[35]^ ATP7B is a copper transporter P1B-type ATPase that maintains intracellular copper ion homeostasis.^[36]^ The expression of FDX1 was positively correlated with the level of infiltration of CD8+ T cells and neutrophils.^[37]^ Inhibition of LIAS expression leads to a decrease in hepatic alpha-lipoic acid levels and an increase in tissue oxidative stress.^[38]^ Moreover, we explored the effects of some common UC drugs on hub CuDEGs to reveal the significance of cuproptosis in active UC. IFX and GLM, which target TNF-α, and VDZ, which target α4β7, are recommended as first-line therapeutic agents for patients with moderate to severe UC. In our study, the expression of ATOX1 was upregulated and the expression of SUMF1, MT1G, ATP7B, FDX1, and LIAS was downregulated in UC patients who responded to GLM after treatment. IFX significantly changed the expression levels of SUMF1, MT1G, ATOX1, FDX1, and LIAS but did not affect ATP7B. The expression patterns of hub CuDEGs before and after VDZ treatment in patients with active UC were similar to those of the GLM. In summary, we verified that the 3 UC drugs have different effects on cuproptosis-related hub genes and modify their expression while alleviating active UC. The association between cuproptosis and active UC is worthy of further in-depth investigation.

We investigated gene expression data from patients with inactive UC and those with active UC using 2 datasets (GSE53306 and GSE75214), and obtained 9157 DEGs. GO enrichment analysis revealed the enrichment of the positive regulation of cytokine production. Cytokines influence cellular interactions in a specific manner. For example, the expression levels of cytokines, such as IL-1β, IL-6, and TNF-α, are relatively high in the colonic tissue of patients with active UC. In our study, DEGs were found to be involved in the MAPK, Rap1, JAK-STAT, and TNF signaling pathways. The MAPK signaling pathway is involved in inflammation, cell growth and differentiation, the cell cycle, and cell death.^[39]^ Activated MAPK inhibits claudin-2 expression, resulting in increased intestinal mucosal permeability, release of large amounts of pro-inflammatory factors, and promotion of intestinal epithelial cell death.^[40,41]^ TNF-α is a monokine produced by macrophages and monocytes, which promotes the development of inflammatory responses. When external factors trigger MAPK protein activation, NF-κB is activated and translocated to the nucleus via MAPK phosphorylation with specific substrates, regulating the production of inflammatory factors, such as TNF-α, and exacerbating the inflammatory response.^[42,43]^ Rap1 deficiency results not only in spontaneous colitis in mice, but also in feedback regulation to amplify Treg-mediated inflammatory response.^[44]^ JAKs represent a group of 4 membrane-bound receptors, which mediate the regulation of genes that code for diverse inflammatory proteins via the signal transducer and activator of transcription (STAT) pathway.^[45]^ The JAK/STAT pathway promotes immune cell differentiation, secretion of pro-inflammatory factors, and exacerbates the inflammatory response in UC.^[46]^

Patients with active and inactive UC exhibit different immune cell abundances. Patients with active UC exhibit higher levels of activated dendritic cells and neutrophils, as well as lower levels of CD8+ T cells, regulatory T cells (Tregs), and macrophage M2. The MAPK, JAK-STAT, and TNF signaling pathways have been reported to be critical for B and T cells differentiation.^[47–50]^ This provides further evidence of the importance of immune dysregulation in the development of UC.

Our study has some limitations. First, only bioinformatics analysis was performed in our study, and clinical or experimental verification of CRGs expression levels in active UC is required. In addition, there is still a need for greater numbers of active UC samples to elucidate the diagnostic values of hub CuDEGs, and further research into the relationship between CuDEGs and immune responses is needed.

## 5. Conclusion

We developed a six-gene model of the cuproptosis signature that could be used to accurately diagnose patients with active UC. In our study, the role of cuproptosis in active UC was uncovered for the first time, and we provide additional insights into the underlying molecular mechanisms of active UC heterogeneity.

## Author contributions

**Conceptualization:** Menglong Zou, Ying Zhu, Yin Xu.

**Data curation:** Menglong Zou, Wei Zhang.

**Formal analysis:** Wei Zhang.

**Funding acquisition:** Menglong Zou, Ying Zhu, Yin Xu.

**Methodology:** Menglong Zou.

**Writing – original draft:** Menglong Zou.

**Writing – review & editing:** Ying Zhu, Yin Xu.
